# Temporary materials: comparison of in vivo and in vitro performance

**DOI:** 10.1007/s00784-020-03278-5

**Published:** 2020-06-24

**Authors:** Tuğrul Sari, Aslihan Usumez, Thomas Strasser, Abdurrahman Şahinbas, Martin Rosentritt

**Affiliations:** 1 Faculty of Dentistry, Department of Prosthodontics, Istanbul Aydın University, Istanbul, Turkey; 2 Private Practice, Istanbul, Turkey; 3 Department of Prosthetic Dentistry, Regensburg University Medical Center, D-93042 Regensburg, Germany; 4 Faculty of Dentistry, Department of Prosthodontics, Bezmialem Vakif University, İstanbul, Turkey

**Keywords:** Temporary materials, Provisional materials, In vitro testing, In vivo investigation, TCML, Roughness, Wear, Fracture force

## Abstract

**Objective:**

The aim of this investigation was to compare clinical performance and in vitro wear of temporary CAD/CAM and cartridge crowns. This study is an approach to estimate the influence of in vivo use and laboratory simulation on temporary crowns.

**Materials and methods:**

A total of 90 crowns were fabricated from each temporary CAD/CAM or cartridge material. Also, 10 crowns of each material were clinically applied for 14 days, and 80 identical duplicate restorations were investigated in the laboratory after storage in water (14 days; 37 °C) and subsequent thermal cycling and mechanical loading (TCML, 240.000 × 50N ML, 600 × 5°C/55 °C). After in vivo application or in vitro aging, facture force, superficial wear (mean and maximum), surface roughness (Ra, Rz), thermal weight loss (TGA), and heat of reaction (DSC) were determined for all crowns. Statistics: Bonferroni post hoc test; one-way analysis of variance (ANOVA); *α* = 0.05).

**Results:**

The fracture resistance of the temporary materials varied between 1196.4 (CAD in vivo) and 1598.3 N (cartridge crown in vitro). Mean (maximum) wear data between 204.7 (386.7 μm; cartridge in vitro) and 353.0 μm (621.8 μm; CAD in vitro) were found. Ra values ranged between 4.4 and 4.9 μm and Rz values between 36.0 and 40.8 μm. DSC and TG analysis revealed small differences between the materials but a strong influence of the aging process.

**Conclusions:**

Comparison of in vivo and in vitro aging led to no significant differences in fracture force and wear but differences in roughness, DSC, and TGA. SEM evaluation confirmed comparability. Comparison of CAD/CAM and cartridge temporary materials partially showed significant differences.

**Clinical relevance:**

In vitro aging methods might be helpful to estimate materials’ properties before principal clinical application. CAD/CAM and cartridge temporary materials provided comparable good clinical performance.

## Introduction

Temporary restorations are regularly required before the delivery of fixed prosthetic restorations. The restorations protect prepared teeth from chemical or thermal influences, maintain function and aesthetics, and shape marginal gingival areas [1, 2]. Temporary restorations can be fabricated with conventional cartridge (two paste) systems, using direct methods on already prepared teeth or indirect methods on laboratory gypsum models. However, contemporary CAD/CAM rapid prototyping (milling, 3D printing) seems to be an innovative alternative fabrication method. Advantages of CAD/CAM fabrication might be the easy availability of patient-specific data, the manufacturing of new restorations in case of loss or fracture, and the use and easy adaptability for bite elevation therapy. Temporary light-curing- or auto-polymerizing resin-based materials consist of polymethyl-methacrylate and ethyl-methacrylate resins, polyvinyl-methacrylate resins, composite-based resins, or urethane-based resins [3, 4]. In individual cases, inorganic fillers are supplemented. Contemporary two component auto-mixing cartridge materials are state of the art and feature good clinical performance [5, 6]. A number of studies addressed the material properties of temporary restorations such as flexural strength, fit, fracture strength, or temperature of reaction [4, 7–12].

CAD/CAM blanks are fabricated under controlled industrial conditions and feature improved mechanical properties, reduced residual monomers, and—due to the milling fabrication—show no heat of reaction. Polymerization shrinkage of temporary cartridge crowns might be a significant factor for marginal discrepancy. This is no issue for CAD/CAM materials, because they are polymerized during fabrication process prior to machining [8]. It was shown that CAD/CAM temporary restorations show superior mechanical properties compared with their conventional counterparts [7]. However, new materials require extensive analyses, including simulation of aging processes and mechanical stability. Important clinical factors influencing the performance of the temporary restorations might be strength (to avoid fracture), wear resistance (to maintain adjusted occlusal situation and avoid crack development), and low water uptake (to avoid discoloration and assure dimensional stability). In vitro tests may be helpful for estimating the principal usability, but finally only in vivo investigations confirm that the materials fulfill the clinical requirements. It has been shown that thermal cycling and mechanical loading can predict clinical failures in laboratory studies, but it seems essential to establish an appropriate validation for individual materials [13]. Temporary restorations might be an ideal substrate for investigating and validating artificial aging, because the restorations can be easily removed after clinical application without damage, allowing the restoration to be analyzed in the laboratory and compared with in vitro specimens. Therefore, the aim of the study was to compare fracture strength, wear, and roughness of temporary restorations under clinical and in vitro conditions. The hypotheses of this study were as follows:

- CAD/CAM and cartridge materials show comparable performance.
- In vitro results are comparable with in vivo data.

## Materials and methods

Applying temporary CAD/CAM (Telio CAD, Ivoclar Vivadent, Schaan, Liechtenstein) and cartridge material (C&B Plus, Ivoclar Vivadent, Schaan, Liechtenstein), 90 crowns were fabricated from each material. Further, these 90 crowns were divided in two groups. Ten crowns were clinically applied for 14 days, and 80 identical duplicate restorations were investigated in the laboratory. Between July 2017 and August 2018, patients of the Department of Prosthodontics, Bezmi Alem Vakıf University, İstanbul, Turkey (Ethical confirmation: Etik Kurul Kararı Sayı: 71306642-050.01.04-) who required prosthetic crown treatment for mandibular first molar teeth were included in the study. A digital randomization table was prepared via a randomization software (www.random.org, Randomness and Integrity Services Ltd., Dublin, Ireland), and the patients were grouped into the conventional or CAD-CAM temporary restoration groups according to the randomization table. Patients were supplied with temporary crowns subsequent to shoulder preparation of the abutment teeth. Excluded from testing were heavy smokers and patients with heavy bruxism. Two commercially available resin-based materials (*n* = 10 per group, materials are listed in Table 1) were used for fabrication of temporary crowns.

**Table 1:** Materials

Trade name	Composition	Group	Shade/LOT	Manufacturer
Telio CAD	99.5% PMMA	CAD/CAM	A2/V16925	Ivoclar Vivadent, Schaan, Liechtenstein
C&B Plus	Two component auto-mixing cartridge bismethacrylate	Cartridge	A2/V01704	

Conventional additional silicone impressions (Variotime Putty and Variotime Light Flow, Kulzer GmbH, Hanau, Germany) and digital impressions (Cerec Omnicam, Sirona, D) were taken in all cases before and after crown preparation, after final insertion, and before removal of temporary crowns. Crown preparations were performed by the same operator using a diamond bur kit (Athen Preparation Crown and Inlay Kit, Hager and Meisinger GmbH, Neuss, Germany). All temporary crowns were prepared according to the manufacturer’s recommendations. Cartridge temporary crowns were fabricated by direct method using the over-impression technique. CAD/CAM-fabricated temporary crowns were fabricated after intra-oral scanning, design, and milling (Cerec Omnicam, MCXL, Sirona, D). All crowns were finished and polished (rotary rubber cups, Astropol Polishing Kit (LOT: UL0405), Ivoclar Vivadent, Schaan, Liechtenstein). Temporary cementation was performed with a dual-cure temporary resin cement (Telio CS Link (LOT: V0794), Ivoclar Vivadent). After clinical service of 2 weeks, all restorations were removed and visually investigated in detail. Restorations were evaluated by the same dentist for fracture in the cusps or fracture in the margins. In all cases, there was no failure of the clinical specimens in terms of visual fracture or cracking.

For the laboratory investigation, temporary crowns served as templates for the fabrication of duplicates from the corresponding cartridge material. The corresponding master cast models and the impressions/molds that had been used for the fabrication of the original temporary restorations (clinical template) were used. Identical CAD/CAM crowns were milled using the digital design for clinical specimens. Eight duplicate temporary crowns were fabricated from each clinical template. Temporary crowns were used to fabricate resin teeth (Palapress Vario, Kulzer GmbH) with the corresponding individual preparation. Resilience of the human periodontium was simulated by coating the roots of the resin teeth with a 1 mm layer of polyether impression material (Impregum, 3M Oral Care, USA) [14]. Coated artificial teeth were finally fixed in resin blocks (Palapress Vario, Kulzer GmbH). Corresponding to the design and procedure of the clinical situation, temporary cementation was performed with the same temporary resin cement (Telio CS Link, Ivoclar Vivadent).

All temporary crowns were stored in distilled water for 14 days (37 °C) corresponding to the time of clinical service. Additional thermal cycling (TC, 2 × 600 cycles between 5 and 55 °C; distilled water) and mechanical loading (ML, 50 N for 240.000 cycles; *f* = 1.6 Hz; mouth opening, 2 mm) were performed to simulate 2 years of clinical service (TCML chewing simulator EGO, Regensburg). Steatite spheres (diameter, 3, 6, 8, or 12 mm; CeramTec, D) were individually positioned to match the clinical occlusal contact situation.

Crowns (after clinical service) and crown duplicates (after storage and TCML) were investigated for surface deterioration (wear, roughness, SEM), stability (fracture test), and water sorption/composition (differential scanning calorimetry (DSC), thermal gravimetric analysis (TGA)). Worn surface areas were digitalized with a 3D laser scanning microscope (KJ 3D, Keyence, J). Mean and maximum wear depths (μm) and surface roughness (Ra, Rz) were determined within the wear facet. The temporary crowns were investigated with scanning electron microscopy (SEM Quanta, Philips, NL). All crowns and duplicates were loaded to fracture (1446, Zwick, D; *v* = 1 mm/min). The load was applied with a steel sphere (*d* = 12 mm) in the center of the crown. A tin foil (0.25 mm, Dentaurum, D) between crown and sphere was used to minimize force peaks. Both temporary materials were investigated for aging effects using differential scanning calorimetry (DSC 204 F1; 25–300 °C, 20 K/min; *n* = 2; Netzsch, Selb, G) and thermal gravimetric analysis (TGA 209 F3; 20–600 °C, 20 K/min, N_2_-atmosphere; n = 2; Netzsch, G) after fabrication (baseline), 14 days in water at 37 °C + TCML (in vitro), and clinical service (in vivo). Calculations and statistical analysis were performed using SPSS 25.0 for Windows (SPSS Inc., Chicago, IL, USA). Means and standard deviations were calculated with Bonferroni post hoc test (*α* = 0.05) and analyzed using one-way analysis of variance (ANOVA).

## Results

**Fig. 1:**
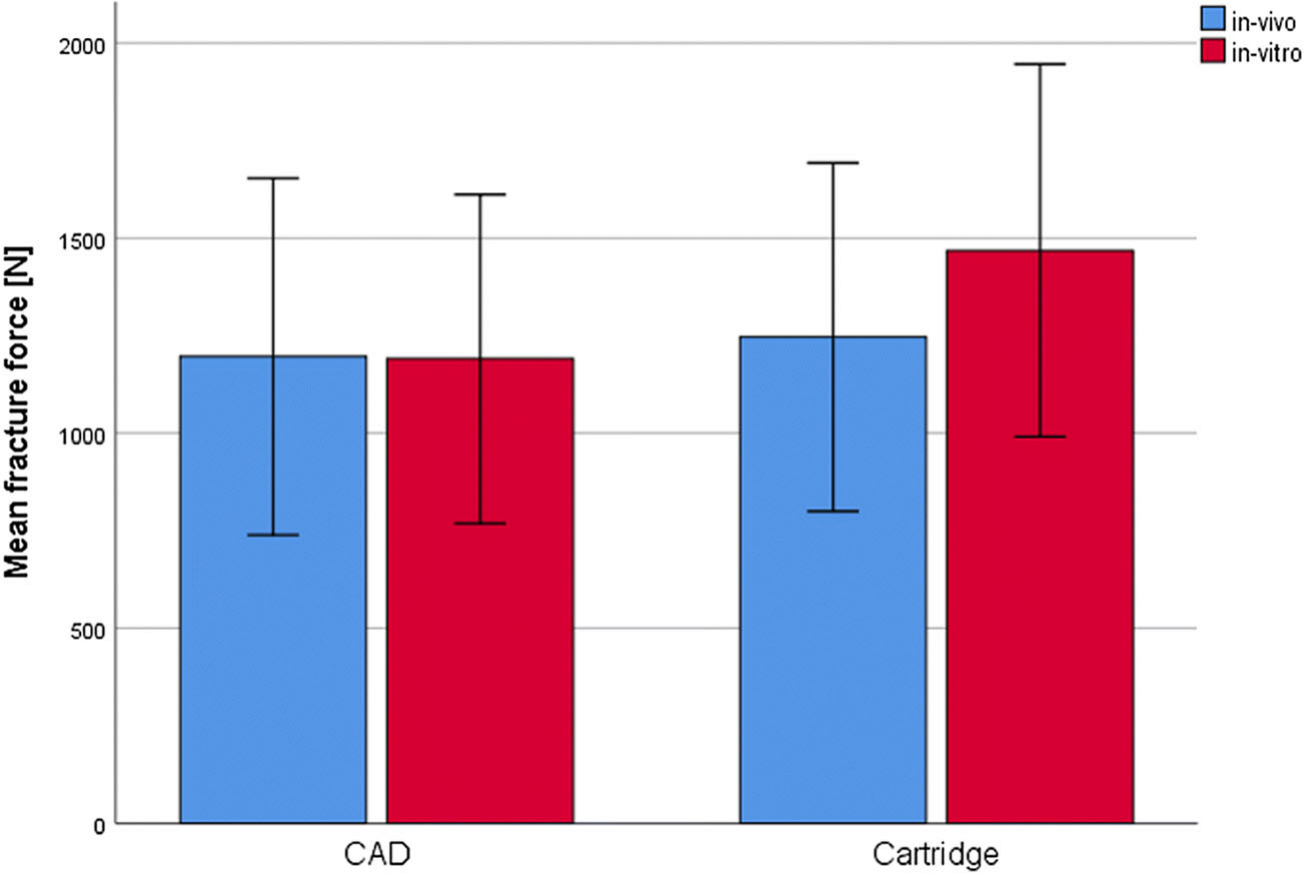
Fracture force [N] of crown materials (CAD/CAM and cartridge) under in vivo or in vitro conditions

**Fig. 2:**
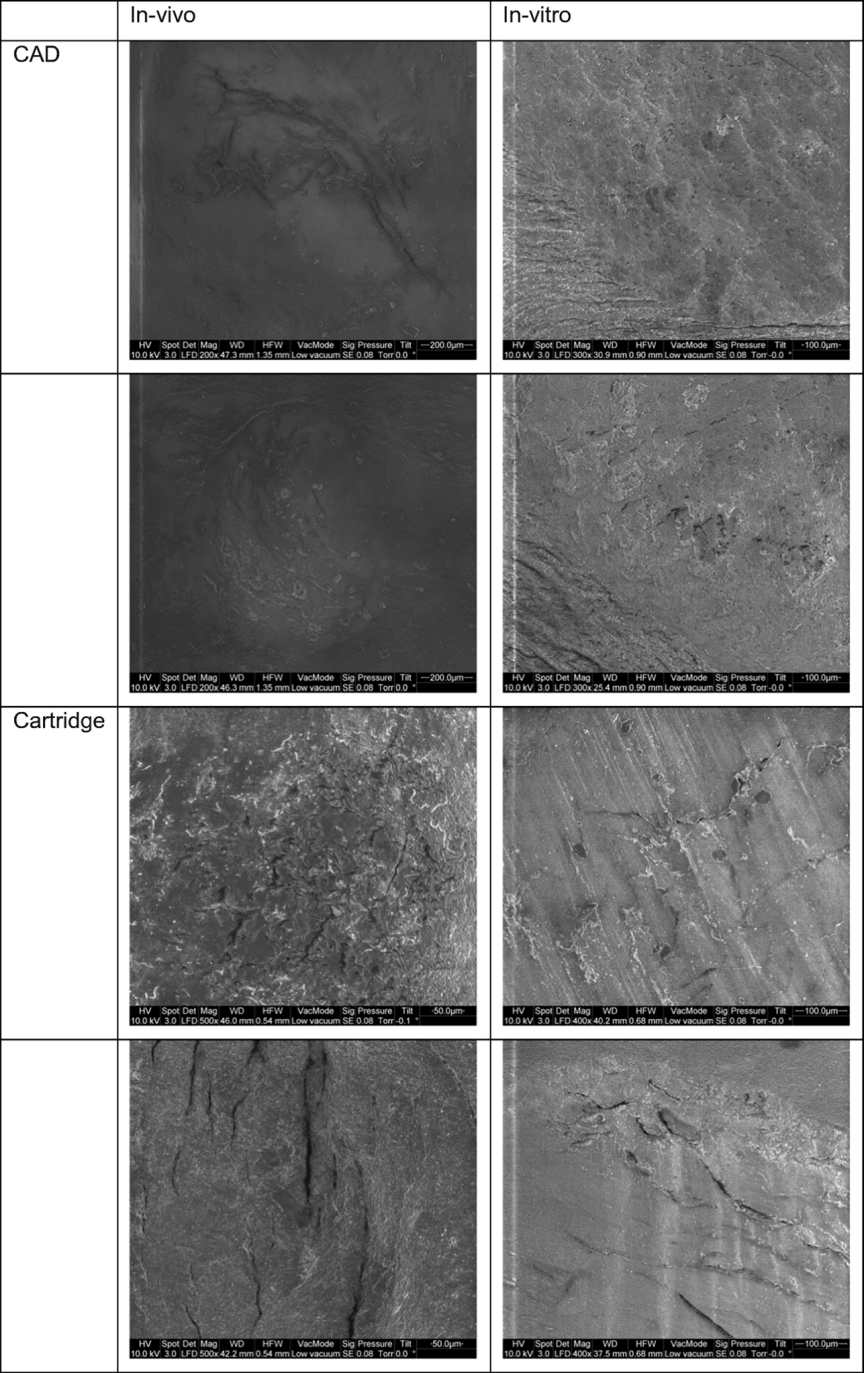
Scanning electron microscopy (SEM, magnification × 200–500, 10 KV, vacuum mode) comparison of wear at contact areas of randomly chosen CAD/CAM and cartridge crowns after in vitro testing and in vivo application

**Fig. 3:**
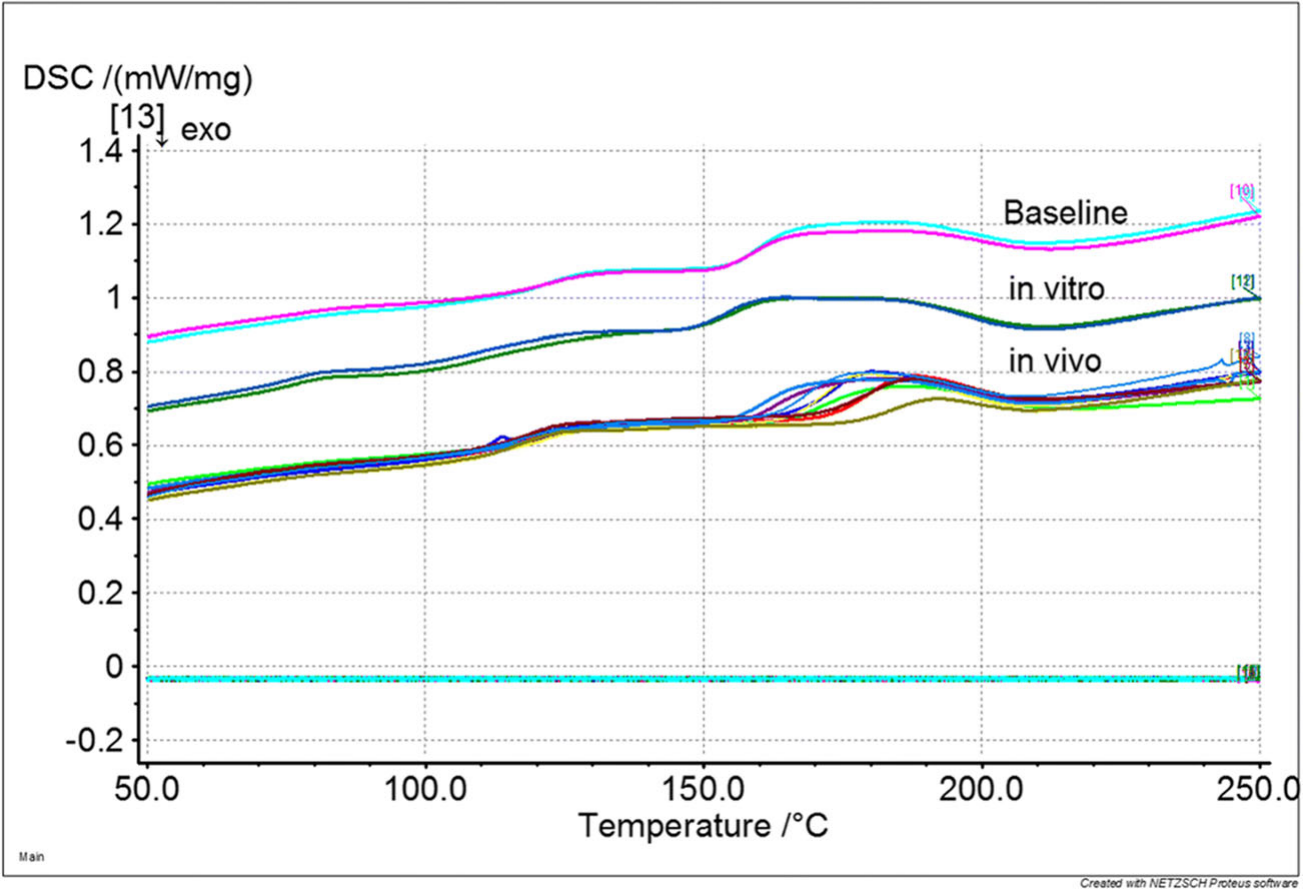
DSC heat flow [mw/MG] (comparison between CAD/CAM and cartridge system; baseline, in vivo, in vitro)

**Fig. 4:**
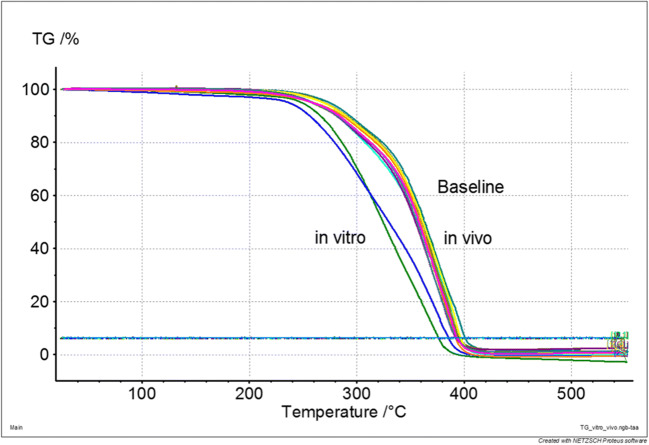
Weight loss [%] with thermal gravimetry (TG, comparison between CAD/CAM and cartridge system (baseline, in vivo, in vitro))

A total of 10 CAD/CAM and 10 cartridge crowns were investigated. None of the crowns failed during in vivo application or in vitro storage and TCML. Facture force: under in vivo conditions, the cartridge material showed no statistically significant (*p* = 0.807) differences compared with the CAD material (CAD, 1196.4 ± 457.4 N, Cartridge, 1246.5 ± 446.7 N). Under in vitro conditions, a significant (*p* = 0.017) difference between CAD (1358.6 ± 520.9 N) and cartridge crown (1598.3 ± 713.0 N) was found. The differences between in vitro and in vivo situations were not statistically significant (CAD, *p* = 0.350; cartridge, *p* = 0.133) for both materials (Fig. 1). Wear: individual in vivo wear traces varied between 2 and 3 contacts. Mean wear was in a range of about 300–350 μm after clinical application and about 200–250 μm after TCML. Mean wear differences between CAD and cartridge were not significant (*p* = 0.237) in vivo, but under in vitro conditions, significantly different (*p* = 0.000) results were found. Mean wear differences between in vitro and in vivo were significant (*p* = 0.036) for the CAD material, but not for the cartridge system (*p* = 0.289; Table 2). Maximum wear varied between 390 and 450 μm for the cartridge and around 615–620 μm for the CAD material. Maximum wear differences between CAD and cartridge were not statistically significant (in vivo: *p* = 0.027; in vitro: *p* = 0.000). Differences between in vitro and in vivo were not significant for both materials (CAD: *p* = 0.888; cartridge: *p* = 0.310) (Table 2). Roughness Ra: under in vivo conditions, no significant (*p* = 0.982) Ra differences were found between CAD and cartridge material, but TCML caused significant (*p* = 0.038) Ra differences between CAD and cartridge systems. Compared with in vivo results, the Ra results after TCML were significantly (*p* < 0.005) different for both materials. Roughness Rz: under in vivo conditions, no significant (*p* = 0.798) Rz differences were found between CAD and cartridge material, whereas under TCML conditions, the results were significantly (*p* = 0.018) different. TCML and in vivo results were significantly (*p* = 0.036) different for the CAD material, but not for the cartridge system (*p* = 0.969) (Table 3). SEM comparison: exemplary pictures confirmed the comparability of in vivo and in vitro wear traces. Both materials provided cracks and material shifting in the contact areas. Surface damage seemed more distinct for the cartridge material (Fig. 2). DSC analysis: the baseline measurements of heat flow provided comparable DSC curves for both materials, indicating only small differences between CAD/CAM and cartridge systems. Exemplary results for the CAD system showed differences between new, in vitro, and in vivo specimens. Compared with the baseline measurement, an additional peak at around 60 °C and a shift of endothermal peaks in the area of about 120 °C and 180 °C were found after in vitro aging. Measurements after clinical application indicated a peak shift at about 120 °C and 180 °C, which was less pronounced in comparison with the in vitro situation (Fig. 3). TGA investigation: measurements indicated a clear weight loss for both materials at around 250 °C. Residual weight at 400 °C was 0 w% for both materials, showing that the materials had no inorganic components. The results for both systems exposed only small differences between baseline and in vivo measurements. Specimens which were investigated after in vitro application (14 days water at 37 °C and TCML) provided a soft weight decrease at around 100 °C and a significantly steeper decrease of the weight curve for both materials in comparison with baseline or in vivo measurements (Fig. 4).

**Table 2:** Mean and maximum wear [μm] (mean ± standard deviation) of crown materials (CAD/CAM and cartridge) under in vivo or in vitro conditions (Bonferroni post hoc test (*α* = 0.05; *p*, probability comparison between in vitro and in vivo)

μm	CAD		Cartridge	
	In vivo	In vitro	In vivo	In vitro
Mean wear	− 296.9 ± 138.1	− 353.0 ± 113.6	− 244.4 ± 169.0	− 204.7 ± 147.3
	*p* = 0.036		*p* = 0.289	
Max. wear	− 615.3 ± 260.3	− 621.8 ± 190.1	− 447.5 ± 244.4	− 386.7 ± 243.0
	*p* = 0.888		*p* = 0.310

**Table 3:** Roughness Ra and Rz [μm] (mean ± standard deviation) of crown materials (CAD/CAM and cartridge) under in vivo or in vitro conditions (Bonferroni post hoc test (*α* = 0.05; *p*, probability comparison between in vitro and in vivo)

μm	CAD		Cartridge	
	In vivo	In vitro	In vivo	In vitro
Ra	4.4 ± 1.6	4.9 ± 1.6	4.4 ± 2.0	4.4 ± 1.8
	*p* = 0.049		*p* = 0.038	
Rz	36.7 ± 12.3	40.8 ± 12.0	36.0 ± 16.9	36.1 ± 12.4
	*p* = 0.036		*p* = 0.969

## Discussion

The hypotheses of this study that CAD/CAM and cartridge materials show comparable performance could be confirmed only in parts. Fracture force, wear results, and surface degradation (SEM) were comparable between in vitro and in vivo data, whereas roughness, DSC, and TGA data partly showed different results.

The fracture results indicated a clear comparability between CAD/CAM and cartridge materials. In contrast to expectations [8, 15, 16], the CAD/CAM system showed no higher fracture results or better reliability (standard deviation) in comparison with the cartridge system. Nevertheless, our results are limited by the high deviation of the results, which might be decisively caused by the diversity of the individual in vivo crown designs. Fracture results of the crowns were within a range, which has previously been found for different temporary materials (711 N-1392 N) [9]. Fracture results are strongly dependent on the crown design. Thus, values for a right first premolar were significantly lower [7]. Considering that the fracture values exceeded maximum chewing forces in the posterior region of about 900 N [17], all groups have the potential to withstand clinically occurring forces. None of the investigated crowns failed during clinical or in vitro application. Fracture results were sufficiently high, although cyclic loading during application might cause strength reduction [9, 18]. Failure patterns such as crown fracture and chipping indicated contact-induced failure in all materials. However, it has to be kept in mind that loading to fracture may not reflect any clinically observable failure modes.

Temporary restorations protect the prepared teeth and maintain function. Wear might be a clinical problem especially for long-term temporary applications, because excessive wear might reduce and change occlusion, function, and stability of the restoration. Wear is a complex phenomenon [19], and measurements were influenced by complex in vivo wear traces, which were found in our clinical situations with 2–3 individual contacts. Copying and transferring the in vivo contact situation to the in vitro test proved to be difficult. Moreover, the elaborate simulation of the in vivo contact situation caused the loss of the in vitro advantage of simple and standardized (contact) conditions. Simplification of the in vitro contact—for example with only one steatite sphere contact—may be required for an easier and substantial evaluation. Thermal cycling and mechanical loading were adjusted to simulate 2 years as a compromise of manageable effort and providing evaluable data. Up to now, no correlation between in vivo and in vitro simulation is provided for temporary materials, which might be used for the testing design. There is only limited information regarding fiber-reinforced or ceramic materials. In vitro simulation of the clinical application time might not have provided evaluable data. The risk of overestimating the materials might thus be avoided by prolonged in vitro simulation of 2 years. Wear results differed between in vivo and in vitro situations, but only the mean results for the CAD/CAM material were different between in vivo and in vitro evaluation. Maximum wear depths were comparable between the two situations. The results indicate that mean wear data show a leveling of the individual materials’ properties. Maximum wear data should be favored, because they might be more prone to show differences between the individual materials. Wear depths were in a range of about 300–350 μm after 14 days of clinical application and about 200–250 μm after 240.000 mechanical loadings. The amount of wear for the temporary restorations seemed extremely high compared with annual wear rates of enamel [20] or restorative composites of around 30 μm [21]. Based on the varying results, it seems impossible to adapt the simulation process by varying the number of in vivo loadings.

Absorbed water can affect the materials’ dimensional stability, accuracy, or mechanical properties, which in consequence will reduce the lifetime of the restoration [10, 22, 23]. Water uptake is a critical property of polymers, which may distort soft acrylics, increase the interaction with biological organisms [24, 25], or weaken and plasticize the polymer links by decreasing glass transition temperature [26]. Weight determination with TGA and heat of reaction with DSC proved a clear influence of water storage on the materials. Both materials strongly provided water uptake even after a relatively short time of exposition. Obviously, water storage has a more pronounced effect on the stored materials than the in vivo usage. This might be explained by a continually and direct exposition during water storage in comparisons to saliva exposure in the mouth. DSC and TGA even show no strong differences between CAD/CAM and cartridge materials indicating a comparable matrix composition. The supposed, improved polymerization and different composition of the CAD/CAM material could not be confirmed by DSC or TGA. TGA data showed that no inorganic fillers were added. It is supposed that filler addition might influence handling properties of the cartridge materials [5] and further improve stability and fracture force of the materials.

SEM pictures revealed clear surface effects for both materials. Material shift, cracks, and wear in the contact areas showed a clear deterioration of the materials, supporting their limited indication and wearing period for clinical use. Nevertheless, the effects seemed more pronounced for the cartridge system. An extension of the application time for the temporary CAD/CAM systems might be possible. SEM pictures revealed comparable failures under in vivo and in vitro conditions. The results confirm the necessity of investigating clinically worn or failed restorations for confirming deterioration effects and describing reasons for failure. As expected, especially affected areas were contact points. Differences between in vivo and in vitro results might be attributed to enamel (in vivo) and steatite antagonists (in vitro) and/or the enhanced influence of water storage. Individual surface effects might be supported by the irregular shape and different design of the crowns. This may point out the necessity of using (human) antagonists or at least articulated clinical relevant situations with standardized tooth-shaped antagonists.

Wear and fracture data support the assumption that 50 N in vitro chewing force might be appropriate for comparison with the in vivo data, although it has been supposed that weaker materials may be aged with lower loading and enduring force for achieving relevant simulations [13]. The need to gather patients with an indication for restoration of mandibular first molar that fit the further requirements led to a low but adequate number of specimens. Because of the individual crowns for different patients in clinical application, study design made it necessary to evaluate different crowns. By the means of a low number of in vivo crowns available and the small number of in vitro samples, the correlation between clinical and in vitro failures is limited. The individuality of abutment teeth and crowns expresses in a high variation of the fracture results as it is found under clinical conditions. Further, different patient and functional conditions contribute to an individual in vivo performance. Thus, comparison of clinical applied crowns is constrained as well as comparison of respective duplicate crowns. Comparison of in vitro and in vivo data of identical crown dimensions was provided, as for each clinical applied crown, 8 identical crowns were tested in vitro.

This study is a first approach to compare in vitro and in vivo data of temporary crowns. Standardized laboratory tests may help to exclude individual factors. This is the reason why we tried to find intersections between in vivo and in vitro testing, helping to improve our understanding of the in vitro testing. Being aware of the limitations, we recommend to provide maximum wear data, because these might be of higher informative value than mean wear data. The results show that simulations with 240.000 loadings at 50 N [27] may provide sufficient estimation of aging of temporary restorations. The simulation allows a satisfactory valuation of the temporary materials excluding catastrophic failures of the crowns. A verification of other simulation parameters seems necessary. To our knowledge, these tests are the first to compare and evaluate in vivo and in vitro data, which might encourage further workgroups in such kind of testing as a higher number of clinical data with gradual failure results would be helpful for improving the quality of the actual evaluation.

## Conclusion

Temporary CAD/CAM and cartridge materials showed small differences in fracture force, wear, and roughness. In vivo and in vitro aging led to comparable results in SEM evaluation. No significant differences in fracture force and wear but differences in roughness, DSC, and TGA were found.
