# Correction to: Temporary materials: comparison of in vivo and in vitro performance

**DOI:** 10.1007/s00784-021-04067-4

**Published:** 2021-07-21

**Authors:** Tuğrul Sari, Aslihan Usumez, Thomas Strasser, Abdurrahman Şahinbas, Martin Rosentritt

**Affiliations:** 1 Faculty of Dentistry, Department of Prosthodontics, Istanbul Aydın University, Istanbul, Turkey; 2 Private Practice, Istanbul, Turkey; 3 Department of Prosthetic Dentistry, Regensburg University Medical Center, D-93042 Regensburg, Germany; 4 Faculty of Dentistry, Department of Prosthodontics, Bezmialem Vakif University, İstanbul, Turkey

## Abstract

A Correction to this paper has been published: https://doi.org/10.1007/s00784-021-04067-4

**Correction to** **:** **Clinical Oral Investigations**

**https://doi.org/10.1007/s00784-020-03278-5**

The article “Temporary materials: comparison of in vivo and in vitro performance”, written by Tuğrul Sari, Aslihan Usumez, Thomas Strasser, Abdurrahman Şahinbas and Martin Rosentritt, was originally published Online First without Open Access. After publication in volume 24, issue 11, page 4061–4068 the author decided to opt for Open Choice and to make the article an Open Access publication. Therefore, the copyright of the article has been changed to © The Author(s) 2020 and the article is forthwith distributed under the terms of the Creative Commons Attribution 4.0 International License, which permits use, sharing, adaptation, distribution and reproduction in any medium or format, as long as you give appropriate credit to the original author(s) and the source, provide a link to the Creative Commons license, and indicate if changes were made. The images or other third party material in this article are included in the article’s Creative Commons license, unless indicated otherwise in a credit line to the material. If material is not included in the article’s Creative Commons license and your intended use is not permitted by statutory regulation or exceeds the permitted use, you will need to obtain permission directly from the copyright holder. To view a copy of this license, visit http://creativecommons.org/licenses/by/4.0.

The original article has been corrected.

